# MicroRNA-199b Deregulation Shows a Strong SET-Independent Prognostic Value in Early-Stage Colorectal Cancer

**DOI:** 10.3390/jcm9082419

**Published:** 2020-07-28

**Authors:** Ion Cristóbal, Jaime Rubio, Blanca Torrejón, Andrea Santos, Cristina Caramés, Melani Luque, Marta Sanz-Álvarez, Ruth Alonso, Sandra Zazo, Juan Madoz-Gúrpide, Federico Rojo, Jesús García-Foncillas

**Affiliations:** 1 Cancer Unit for Research on Novel Therapeutic Targets, Oncohealth Institute, IIS- Fundación Jiménez Díaz-UAM, E-28040 Madrid, Spain; jaime.rubiop@quironsalud.es (J.R.); blanca.torrejonmoreno@gmail.com (B.T.); andreasantos.asc@gmail.com (A.S.); ccarames@fjd.es (C.C.); 2 Translational Oncology Division, Oncohealth Institute, IIS- Fundación Jiménez Díaz-UAM, E-28040 Madrid, Spain; 3 Medical Oncology Department, University Hospital “Fundación Jiménez Díaz”, UAM, E-28040 Madrid, Spain; 4 Pathology Department, IIS-Fundación Jiménez Díaz-UAM, E-28040 Madrid, Spain; melani.luque@quironsalud.es (M.L.); marta.sanza@quironsalud.es (M.S.-Á.); ruthalonsoalonso@gmail.com (R.A.); szazo@fjd.es (S.Z.); jmadoz@fjd.es (J.M.-G.)

**Keywords:** miR-199b, SET, early-stage CRC, tumor suppressor, prognosis

## Abstract

The endogenous PP2A inhibitor SET Nuclear Proto-Oncogene (SET) has been reported to play oncogenic roles and determines poor outcomes in colorectal cancer (CRC). Our group previously showed that miR-199b is deregulated in metastatic CRC, and reduced the cell viability and enhanced the sensitivity of CRC cells to standard induction chemotherapy drugs, mainly through direct negative SET regulation. Clinically, miR-199b downregulation was identified as the molecular mechanism responsible for SET overexpression in around half of metastatic CRC patients. However, the potential clinical value of miR-199b in early-stage CRC remains totally unknown. Thus, here we explored the expression levels of this microRNA in a cohort of 171 early-stage CRC patients using real-time polymerase chain reactions. MiR-199b downregulation was found in 21.6% of cases (37 out of 171) and was significantly associated with those patients with a worse Eastern Cooperative Oncology Group (ECOG) status (*p* = 0.045). Moreover, miR-199b downregulation predicted shorter overall (*p* < 0.001) and progression-free survival (*p* = 0.015). As expected, we next immunohistochemically analyzed SET, observing that it was significantly associated with miR-199b in our cohort. However, multivariate analyses showed that miR-199b was an independent biomarker of poor outcomes in early-stage CRC with a predictive value stronger than SET. In conclusion, our results highlight the potential clinical usefulness of miR-199b and suggest that it could represent a novel molecular target in this disease.

## 1. Introduction

Colorectal cancer (CRC) represents the most frequent gastrointestinal cancer and one of the major causes of morbidity and mortality worldwide; it is the second-most common cause of cancer-related deaths, with around 900,000 deaths annually. The pathology of CRC involves both genetic and epigenetic events, together with environmental determinants, such as dietetic habits, which all contribute to the development of this disease. [1]. The stage of the disease at diagnosis represents a critical relevant prognostic factor, and those cases with metastatic development still have very poor outcomes [2,3]. Thus, more than 70% of newly diagnosed CRC patients have a surgically resectable and not a metastatic disease at the beginning [4,5], but only 15–25% of cases with liver metastases are candidates for surgery [6]. Therefore, it is necessary to better understand the molecular basis of this disease to anticipate and prevent metastatic progression [7].

MicroRNAs (miRs) are a class of single-stranded and small non-coding RNAs (18 to 25 nucleotides) that posttranscriptionally inhibit specific genes. This inhibition is due to the sequence complementarily binding through the “seed region” of the miR, consisting of 7–8 nucleotides located at the 5’ end, to the 3´ untranslated region (3´UTR) of the mRNA target, and leading to its degradation or its transcriptional blocking. Their roles in human cancer have been largely reported since miRs function as regulators of signaling pathways that are crucial for the tumor cell and are involved in proliferation, apoptosis, or cell differentiation. In fact, depending on their specific targets, miRs could act as oncogenes or tumor suppressors [8]. Although miR-199b has been largely reported as a tumor suppressor that is commonly downregulated in several types of human cancers [9,10,11,12,13,14,15,16,17,18,19], only a few studies about miR-199b in CRC have been published to date. In concordance with the tumor suppressor role reported for miR-199b in other tumor types, miR-199b downregulation was found to contribute to CRC progression through the modulation of the SIRT1/CREB/KISS1 pathway via SIRT1 activation [20]. Moreover, circNSD2 has been reported to target miR-199b, thereby leading to the activation of DDR1/JAG1 signaling, which promotes the development of CRC metastatic disease [21]. MiR-199b has been shown to directly target SET Nuclear Proto-Oncogene (SET) in choriocarcinoma [22], and our group demonstrated that SET is also a direct target of this miR in CRC. MiR-199b downregulation led to the reactivation of the tumor suppressor PP2A, which reduced cell growth and enhanced the sensitivity to oxaliplatin in CRC cells. In addition, miR-199b downregulation was predictive of poor outcomes in metastatic CRC and was proposed as the molecular event responsible for SET deregulation in around half of SET-overexpressing patients [23]. Finally, two recent works have analyzed the role of miR-199b in locally advanced rectal cancer (LARC). Of note, Baek and colleagues reported that high levels of both tissue and exosomal miR-199a/b would be correlated with better responses to chemoradiotherapy in locally advanced rectal cancer [24]. Moreover, our group confirmed that miR-199b downregulation has a predictive value regarding responses to neoadjuvant chemoradiotherapy (CRT) in LARC. This observation was probably due to the deregulation of the miR-199b/SET signaling axis since both miR-199b and SET were found to regulate the 5-FU resistance phenotype [25]. However, it remains to be determined whether miR-199b expression levels also have clinical and therapeutic relevance in the subgroup of early-stage CRC patients.

In the present work, we analyzed for the first time the potential clinical value of miR-199b deregulation in CRC patients without metastatic disease at diagnosis. MiR-199b was quantified in a cohort of 171 CRC patients with localized disease and we found that miR-199b downregulation was a recurrent molecular event that strongly determined poor overall (OS) and progression-free survival (PFS) in this disease in a SET-independent manner.

## 2. Materials and Methods

### 2.1. Patient Samples

All the samples used in this research study were kindly supplied by Fundacion Jimenez Diaz Biobank (Madrid, Spain) (PT13/0010/0012). This work included a cohort of 171 consecutive formalin-fixed, paraffin-embedded (FFPE) tumor samples from CRC patients without metastatic disease at diagnosis, and selected from 2001 to 2014 in a retrospective manner. The selection criteria were as follows: adenocarcinoma, absence of neoadjuvant therapy, operable disease, enough material, clinical follow-up data available, and lack of metastatic disease. TNM (tumor, node, metastases) staging was performed based on the 7th American Joint Committee on Cancer (AJCC) staging system established for CRC. Clinical information from each patient was obtained from medical records by two oncologists involved in this study (JR and CC). We performed tissue microarrays (TMA) by carefully selecting representative areas of each tumor sample and obtaining three tissue cores of 1 mm diameter each by using a TMA workstation (T1000 Chemicon). All specimens were taken anonymously and both the Ethical Committee and Institutional Review Board of the Fundacion Jimenez Diaz Hospital evaluated and approved the project (reference number 16/18). Written informed consent was obtained in all cases for the use of biological samples for research purposes.

### 2.2. Nucleic Acid Isolation

Isolation of total RNA from the FFPE tumor samples was carried out using the RecoverAll Total Nucleic Acid Isolation Kit (Thermo Fisher Scientific, Waltham, MA, USA) according to the manufacturer’s recommendations. The FFPE tumor sections were obtained from the interior of the paraffin block to avoid potential contaminations or nucleic acid damage during their storage. For the RNA isolation procedure, we used two consecutive 10 µm sections from the tumor blocks of each patient cut in a microtome. The sections were included in positively charged glass slides and tumor areas were carefully selected by a pathologist. Next, we removed the non-tumor circundant tissue before starting the nucleic acid isolation. Briefly, tumor tissue was included in a 1.5 mL tube and 1 mL of xylene was added to remove the paraffin from the sample; then, the tissue was vortexed and incubated for 30 min at 60 °C before being centrifuged at maximum speed for 2 min at room temperature to pellet the tumor tissue and remove the supernatant that contained the xylene. The next step consisted of two ethanol washes to totally remove the xylene from the samples. Next, 1 mL of ethanol is added to each sample, followed by 2 min of incubation and centrifugation at maximum speed, and the tumor tissue turned opaque. The ethanol was removed after the second wash and the sample was dried at 40 °C for 20 min. Then, 100 µL of digestion buffer and 4 µL of protease was added per sample, mixed gently with a pipette, and incubated at 50 °C for 15 min, followed by another incubation at 80 °C for 15 min. Next, 120 µL of isolation additive and 295 µL of ethanol was added to each sample and mixed by pipetting up and down. A filter cartridge was placed in one of the supplied collection tubes and the sample mixture was applied to the filter cartridge, centrifuged at 10,000× *g* for 30 s, the flow-through was discarded, and the filter cartridge was re-inserted into the same collection tube. Then, 700 µL of wash 1 was added to the filter cartridge and centrifuged at 10,000× *g* for 30 s, and the flow-through was discarded. Next, 500 µL of washes 2 and 3 were added to the same filter cartridge, centrifuged at 10,000× *g* for 30 s, the flow-through was discarded, and the Filter cartridge was re-inserted into the same collection tube. An additional centrifugation was performed at 10,000 × *g* for 30 s to remove residual washing fluids from the filter. For nuclease digestion, 60 µL of the following mix was added to the center of the filter cartridge of each tissue sample: 6 µL 10× DNase buffer + 4 µL DNase + 50 µL nuclease-free water, and then incubated for 30 min at room temperature. Next, 700 µL of wash 1 was added to the filter cartridge and centrifuged at 10,000× *g* for 30 s, and the flow-through was discarded. Then, 500 µL of washes 2 and 3 were added to the same filter cartridge, centrifuged at 10,000× *g* for 30 s, and the flow-through was discarded. The steps with washes 2 and 3 were repeated a second time. After the last centrifugation, the filter cartridge was transferred to a clean collection tube. Next, 40 µL of the elution solution was applied to the center of the filter cartridge, incubated for 2 min at room temperature, the cap was closed, and the sample was centrifugated at 10,000 × *g* for 1 min. The elution was conserved but the last step was repeated with the other 20 µL of the elution solution. Finally, the concentration of the total isolated RNA was determined by measuring its absorbance at 260 nm in a NanoDrop Spectrophotometer (Thermo Fisher Scientific, Waltham, MA, USA).

### 2.3. Quantification of miRNA Expression Levels

Taqman MicroRNA Assays are highly selective since they are delivered in two tubes containing an miR-specific stem-loop reverse transcription (RT) primer to produce a template for the real-time PCR and the PCR primer set, respectively. The samples included in this study were reverse transcribed using the TaqManHMicroRNA Reverse Transcription Kit (Applied Biosystems, Foster City, CA, USA). The RT reaction mix included the following in each sample: 6 µL of RT primer pool, 0.30 µL of dNTPs with dTTP (100 mM), 3 µL of MultiScribe reverse transcriptase (50 U/µL), 1.50 µL of 10× RT buffer, 0.19 µL of RNase inhibitor (20 U/µL), and 1.01 µL of nuclease-free water. The mix was prepared on ice with a total volume of 12 µL per sample and inverted several times to mix it properly; then, it was centrifuged to collect all the components at the bottom of the tube. Next, 3 µL of total RNA at a concentration of 1 to 20 ng/µL was added and the reaction was sealed in a 96-well plate or capped tubes, centrifuged to mix all the components, and incubated for 5 min at room temperature. The tubes or 96-well plate was placed into a thermal cycler and the following reaction conditions were applied: 30 min at 16 °C, 30 min at 42 °C, 5 min at 85 °C, and held at 4 °C until the reaction stopped. Next, the quantification of mature miRNAs was carried out using a quantitative real-time reverse transcription polymerase chain reaction (RT-PCR) with TaqMan MicroRNA Assays (Applied Biosystems, Foster City, CA, USA), which are specific for miR-199b (reference number: miR-199b-5p_000500). U6B was used as a housekeeping gene for data normalization. The PCR reaction mix was prepared in a total volume of 10 µL by adding the following components: 0.08 µL of cDNA from the RT product obtained in the previous step, 0.5 µL of 20× TaqMan MicroRNA Assays, 5 µL of PCR Master Mix, and 4.42 µL of nuclease-free water. The samples were inverted several times to mix them properly and they were centrifuged briefly to collect all the reaction components at the bottom of the tube. Next, 10 µL of the PCR reaction mix was added to each well of a 96-well plate. The plates were then sealed using MicroAmp Optical Adhesive Films (Applied Biosystems, Foster City, CA, USA), mixed, and centrifuged to collect all the reaction components at the bottom of the wells. The plate was placed into a thermal cycler and the amplification reaction was initiated. All the amplification reactions were carried out in an Applied Biosystems 7500 Sequence Detection System. The conditions for each amplification assay were: 95 °C for 10 min for enzyme activation, followed by 45 cycles of 95 °C for 15 s (denature), and 60 °C for 1 min (anneal and extend). The relative gene expression data were analyzed using the 2^−ΔΔ*C*T^ method, as previously described [25], where ΔΔ*C*T = (*C*T,Target Gene − *C*T,U6B)Tumor − (*C*T,Target Gene − *C*T,U6B) Control. We considered miR-199b downregulation had occurred when its expression levels in a specific patient sample were lower than the mean value of miR-199b expression minus standard deviation (SD) in the entire patient cohort as previously described [25].

### 2.4. Immunohistochemistry

Positively charged glass slides were used to include tumor tissue sections that were 3 μm in size. Tumor specimens were then deparaffinized using xylene and graded alcohols, and heat antigen retrieval was carried out in a Dako EDTA-based buffer with a pH of 9 (Agilent, Santa Clara, CA, USA). The blocking of endogenous peroxidase was performed via incubation in 0.03% hydrogen peroxide for 5 min. Next, the slides were incubated with a specific rabbit polyclonal anti-SET (Abcam, Cambridge, UK), as previously described [23], at room temperature for 60 min and followed by incubation with an appropriate anti-Ig horseradish peroxidase-conjugated polymer (Flex+, Dako). 3,3′-Diaminobenzidine was used as a chromogen to visualize the processed tissue sections. All stainings were carried out using a Dako Autostainer. Sections incubated with normal non-immunized rabbit immunoglobulins were used as negative controls. As a positive control, a section of a colorectal tumor with a known expression of SET was used. The SET antibody sensitivity (1:5000) was calculated in a range of crescent dilutions of the primary antibody. Only the membrane of epithelial cells, but not the stromal cells, was evaluated for the SET expression by two pathologists blinded to the clinical data (SZ and FR). Through the estimation of the percentage of tumor cells showing positive staining with low, medium, or high intensity, we calculated a semiquantitative histoscore. To calculate the final score, a weighting factor estimate was applied for each of them. The formula used was as follows: histoscore = (low %) × 1 + (medium %) × 2 + (high %) × 3 and the results ranged from 0 to 300.

### 2.5. Statistical Analysis

The SPSS 20 bioinformatic tool for windows (SPSS Inc, Chicago, IL, USA) was used to perform the statistical analyses. The overall survival (OS) was calculated as the time from the date of diagnosis to the date of death from any cause or the last follow-up. The progression-free survival (PFS) was calculated as the time from the date of diagnosis until any primary, regional, or distant recurrence, the appearance of a secondary tumor, death, or the last follow-up. The time to metastasis (TTM) was calculated as the time from diagnosis until a distant recurrence in those cases with metachronous metastases. Metachronous metastases were considered in those cases who developed distant progression after one year from the date of diagnosis. Kaplan–Meier plots were used to evaluate survival and comparisons were carried out using a log-rank test if the proportional hazard assumption was fulfilled and a Breslow test otherwise. Significant parameters in the univariate analysis were taken into consideration to adjust the Cox multivariate analyses. The cutoff point for miR-199b expression was determined as previously described for metastatic CRC [23]. This work was carried out in accordance with the Reporting Recommendations for Tumor Marker Prognostic Studies (REMARK) guidelines [26]. A *p*-value of less than 0.05 was considered statistically significant.

## 3. Results

### 3.1. Prevalence of MiR-199b Deregulation and Its Association with Molecular and Clinical Parameters in Early-Stage CRC Patients

To evaluate its clinical impact, we measured the expression levels of this miR-199b using real-time PCR in a series of 171 CRC patients without metastatic disease at diagnosis. The patient characteristics of the cohort studied can be found in Table S1. We observed that miR-199b was downregulated in 37 out of 171 cases (21.6%), and this alteration was associated with a worse Eastern Cooperative Oncology Group (ECOG) performance status (45.9% versus 19.7%, *p* = 0.045). Statistical analyses showing the association between molecular and clinical parameters of our cohort and miR-199b expression are included in Table 1.

Next, we evaluated the potential association of miR-199b downregulation with relapse (local or distant), but it was not statistically significant. After stratifying our cohort by stage, we found that this alteration showed a similar and not statistically significant association in all stages (Table S2).

### 3.2. Clinical Significance of MiR-199b Downregulation in Early-Stage Colorectal Cancer

**Figure 1:**
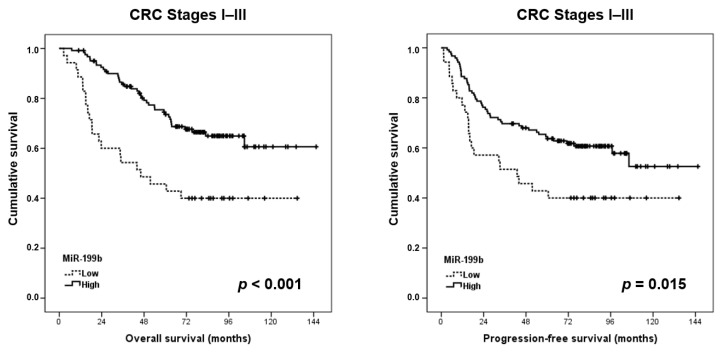
Survival analyses using Kaplan–Meier plots for miR-199b expression in early-stage CRC patients, showing both the overall survival and progression-free survival. CRC, colorectal cancer.

To perform the statistical analyses of survival, we studied a cohort of 157 cases (96 male and 61 female) with available clinical follow-up data; these patients had a median of age of 73 years (range: 38–91) and an OS of 71.5 months. Of importance, a markedly shorter OS (*p* < 0.001) and PFS (*p* = 0.015) were found in those cases with downregulated miR-199b expression (Figure 1). In those cases who developed metachronous metastases (*n* = 44), miR-199b downregulation correlated with a slightly shorter time to metastasis (17.2 vs. 21.2 months), but significance was not achieved (*p* = 0.397) (Figure S1).

**Figure 2:**
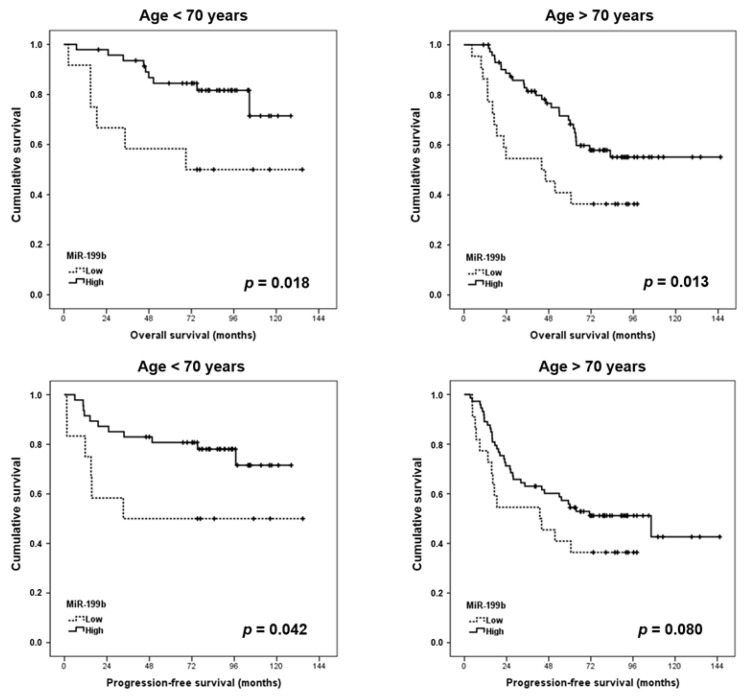
Kaplan–Meier analyses for miR-199b in the cohort of 157 early-stage CRC patients stratified by age.

Next, we studied whether miR-199b downregulation could have clinical relevance after stratifying our cohort into patients younger and older than 60 years. Interestingly, we observed that miR-199b showed a significant prognostic value in both subgroups of younger and elderly patients (Figure 2).

In addition, we analyzed the prognostic value of miR-199b after stratifying our cohort by stage. Although miR-199b downregulation predicted shorter OS in both subgroups of stages I–II (*p* < 0.001) and stage III (*p* = 0.017) patients, significance was only achieved in the subgroup of stages I–II for PFS (*p* = 0.006 vs. *p* = 0.118) (Figure S2).

### 3.3. MiR-199b Correlated with SET but Showed an Independent Prognostic Value

The fact that SET has previously been reported as a key miR-199b target in metastatic CRC prompted us to analyze its expression levels using immunohistochemistry in 144 cases from our cohort. We found SET overexpression in 32 out of 144 cases (15.3%). As expected, we observed that SET overexpression significantly correlated with miR-199b downregulation (*p* = 0.004) (Table 2). For the survival analyses, we included 131 cases with available clinical follow-up data. In concordance with previous studies, we found that SET overexpression determined a significantly worse OS (*p* = 0.020) and PFS (*p* = 0.015) (Figure S3).

However, 22 out of 32 cases with low miR-199b levels showed low SET levels and 12 out of 22 cases with SET overexpression showed high miR-199b levels, indicating that the subgroups of cases covered by these alterations were considerably different. Notably, multivariate analysis demonstrated that miR-199b downregulation was an independent predictor of poor outcome in our cohort in both overall (*p* = 0.002) and progression-free survival (*p* = 0.023), with even higher significance that SET in the case of overall survival. In fact, SET did not reach statistical significance in the multivariate analyses for overall survival (*p* = 0.082). However, SET overexpression was an independent prognosticator in multivariate analyses regarding progression-free survival and with even more significance than miR-199b in this case (*p* = 0.014) (Table 3 and Table S3).

## 4. Discussion

The tumor suppressor miR-199b has been described as being downregulated in many tumor types and it has also been recently proposed as a novel molecular target with therapeutic value [27]. Moreover, miR-199b has been described as showing prognostic value in hepatocellular carcinoma, acute myeloid leukemia, breast and bladder cancers, and metastatic colorectal cancer [23,28,29,30,31]. To explore the clinical impact of miR-199b deregulation in early-stage CRC, we quantified this miR in a cohort of 171 CRC patients without metastatic disease at diagnosis. Of importance, we found that miR-199b downregulation is a molecular event that predicts poor outcomes in this disease (Figure 1). Interestingly, our group previously reported that miR-199b downregulation had a prevalence of 25% in metastatic CRC, which is slightly higher than the 21.8% observed in CRC patients with localized disease. These issues could be explained by the fact that miR-199b downregulation contributes to disease aggressiveness and represents a secondary alteration acquired in the progression of the disease, similar to what has been proposed for SET overexpression [23]. Interestingly, in both studies, the prevalence of miR-199b downregulation was lower in rectal cancer than in colon cancer cases (22% vs. 18.6% in localized CRC and 26.4% vs. 20% in metastatic CRC), but the differences did not reach statistical significance. Moreover, the association between miR-199b and ECOG observed in the present work (*p* = 0.045) (Table 1) was not found in metastatic CRC patients (*p* = 0.175) [23]. In concordance with the results shown here, our group has recently reported that miR-199b downregulation confers shorter overall survival in locally advanced rectal cancer (LARC) patients, which was a subgroup of cases who received preoperative 5-fluorouracil (5-FU)-based neoadjuvant chemoradiotherapy (CRT). We observed that the miR-199b/SET axis plays a key role in determining 5-FU resistance, and both miR-199b and SET predicted the responses to CRT in our LARC cohort [32]. Although SET overexpression has also been described as able to predict worse prognoses in early-stage CRC [33], several differences highlight the prognostic value of miR-199b. This issue is based on the fact that this miR contributes to CRC progression toward the regulation of several other targets besides SET, and that miR-199b was found to be downregulated in around only 50% of cases with metastatic CRC and high SET expression [23]. In fact, here we obtained similar results, where miR-199b downregulation was observed in 10 out of 22 SET-overexpressing patients. Although both alterations were found to be correlated with worse ECOG performance status, SET overexpression was higher in those cases with stage III and the prevalence of miR-199b downregulation was similar in patients with stage III than in those cases with stages I–II (Table 1). Next, we observed a lack of correlation between miR-199b levels and relapse (local or distant) (Table S2), in contrast with the strong SET association with this parameter. In addition, in contrast with our findings here, a high expression of SET significantly determined a shorter time to metastasis, where its prognostic clinical impact was especially marked in those patients older than 60 years [23]. However, we found here that miR-199b downregulation shows prognostic value in both subgroups of younger and elderly patients (Figure 2). Therefore, there were significant differences between miR-199b downregulation and SET overexpression, and the fact that the first one was more prevalent (21.6% vs. 15.3%) further highlights that miR-199b would be including cases with poor outcomes and normal SET levels. It has been recently suggested the relevant value of miR-199b can be used as a prognostic marker in liquid biopsies in a group of 69 LARC patients [24], and it would be very interesting to confirm its potential usefulness in both early-stage and metastatic CRC in forthcoming studies. As SET is a key direct miR-199b target, we evaluated whether the prognostic value of miR-199b was dependent on its association with SET. Notably, we observed in this work that miR-199b was an independent prognosticator with higher significance than SET, probably because miR-199b is a tumor suppressor that regulates additional targets, which strengthens its clinical impact. However, a recent publication showing that SET is a key miR-199b target for mediating 5-FU resistance in LARC [32] indicates that miR-199b could also be regulating additional cell functions observed for SET, such as migration, invasion, and the epithelial to mesenchymal transition, and this issue should be evaluated in future research works. Another relevant issue that remains to be investigated is to determine the molecular mechanisms involved in miR-199b deregulation in CRC. In this regard, miR-199b has been reported to be downregulated through epigenetic silencing in ovarian cancer [13] and long non-coding RNAs in cervical and non-small cell lung cancers [34,35]. In addition, a recent study has investigated the role of the NSD2 circular RNA in the miR-199b inhibition in CRC [21]. However, the identification and clarification of the relevance of these mechanisms that govern miR-199b regulation in cancer, specifically in CRC, remains to be further investigated.

## 5. Conclusions

In conclusion, we found here that miR-199b downregulation was a common event in early-stage CRC that independently predicts worse overall and progression-free survival, thereby defining a subgroup of cases with very poor prognosis. However, the use of a unique cohort of 171 cases represents a relevant limitation of this study such that conclusions have to be taken with caution. Moreover, the widely used RNU6B as a housekeeping gene for data normalization could represent another limitation since its expression was shown to be unstable [36]. Therefore, further validation in forthcoming studies including independent large cohorts, as well as several internal controls, would be required to validate our observations and establish the potential usefulness of this novel biomarker in the clinical routine for early-stage CRC patients.

## Figures and Tables

**Table 1:** Association between miR-199b and clinical and molecular characteristics in 171 colorectal cancer (CRC) patients with localized disease at diagnosis.

Parameters	No. Cases	High MiR-199b (%)		Low MiR-199b (%)		*p*
MiR-199b	171	134	(78.4)	37	(21.6)	
Sex	171	134		37		0.478
Male	107	82	(76.6)	25	(23.4)	
Female	64	52	(81.2)	12	(18.8)	
Age	168	132		36		0.616
<70	62	50	(81.9)	12	(18.1)	
≥70	106	82	(77.4)	24	(22.6)	
ECOG	168	132		36		0.045
0–1	157	126	(80.3)	31	(19.7)	
2–3	11	6	(54.1)	5	(45.9)	
T	171	134		37		0.333
1	3	3	(100)	0	(14.3)	
2	40	34	(85)	6	(15)	
3	108	80	(74.1)	28	(25.9)	
4	19	16	(84.2)	3	(16.8)	
x	1	1	(100)	0	(0)	
*n*	171	134		37		0.848
0	82	64	(78)	18	(22)	
1	46	37	(80.4)	9	(19.6)	
2	30	24	(80)	6	(20)	
x	13	9	(69.2)	4	(30.8)	
Stage	171	134		37		0.924
I–II	82	64	(78)	18	(22)	
III	89	70	(78.7)	19	(21.3)	
Site of primary tumor	166	131		35		0.643
Colon	123	96	(78)	27	(22)	
Rectum	43	35	(81.4)	8	(18.6)	
Metachronous metastasis	171	134		37		0.825
No	127	99	(78)	28	(22)	
Yes	44	35	(79.5)	9	(20.5)	

ECOG, Eastern Cooperative Oncology Group.

**Table 2:** Association between miR-199b and SET in 144 early-stage CRC patients.

	No. Cases	High MiR-199b (%)		Low MiR-199b (%)		*p*
SET	144	112		32		0.004
Low	122	100	(82)	22	(18)	
High	22	12	(54.5)	10	(45.5)	

SET, SET Nuclear Proto-Oncogene; CRC, colorectal cancer.

**Table 3:** Univariate and multivariate Cox analyses in the cohort of 157 patients with early-stage CRC.

	Univariate OS Analysis				Multivariate OS Cox Analysis			
Parameters	HR	95% CI		*p*	HR	95% CI		*p*
		Lower	Upper			Lower	Upper	
Gender				0.576				
Male	1.000							
Female	0.862	0.512 to 1.451						
Age				0.011				0.022
<70	1.000				1.000			
>70	2.147	1.189 to 3.380			2.193	1.120 to 4.293		
Stage				0.015				0.184
I–II	1.000				1.000			
III	1.934	1.137 to 3.287			1.661	0.786 to 3.512		
ECOG				<0.001				<0.001
0–1	1.000				1.000			
2–3	2.274	1.588 to 3.256			2.069	1.388 to 3.085		
T				0.224				
1–2	1.000							
>2	1.217	0.887 to 1.670						
*n*				0.046				0.447
0–1	1.000				1.000			
2–x	1.727	1.009 to 2.954			1.297	0.663 to 2.536		
SET				0.023				0.082
Low	1.000				1.000			
High	2.121	1.112 to 4.046			1.843	0.926 to 3.666		
MiR-199b				0.001				0.002
Low	1.000				1.000			
High	0.399	0.234 to 0.678			0.378	0.207 to 0.693		

OS: Overall Survival; CI: Confidence Interval, HR: Hazard Ratio.

## Supplementary Materials

The following are available online at https://www.mdpi.com/2077-0383/9/8/2419/s1. Figure S1: Clinical impact of miR-199b over time to metastasis. Figure S2: Kaplan–Meier analyses for miR-199b expression in the cohort of 157 CRC cases stratified by stage. Figure S3: Clinical significance of SET in the cohort of 157 early-stage CRC patients. (a) Immunohistochemical images of SET in LARC patients representing low, medium, and high staining. The line shows 25 µm. Original magnification 400×. (b) Kaplan–Meier analyses for SET expression in early-stage CRC patients. Table S1: Clinical and molecular characteristics of a series of 171 CRC patients without metastatic disease at diagnosis. Table S2: Association between miR-199b and progression (local or distant) in 171 stage I–III CRC patients. Table S3: Univariate and multivariate Cox analyses in the cohort of 157 patients with early-stage CRC.
