# Supplementary material for: MicroRNA-199b Deregulation Shows a Strong SET-Independent Prognostic Value in Early-Stage Colorectal Cancer
Source: J Clin Med. 2020 Jul 28;9(8):2419. doi: 10.3390/jcm9082419 (PMC7465379; doi:10.3390/jcm9082419)
Supplement: Supplementary file 1 [file jcm-09-02419-s001.pdf]

Table S1. Clinical and molecular characteristics of a series of 171 CRC patients without metastatic disease at diagnosis.

		No. (%)	
Sex			
	Male	107	(62.6)
	Female	64	(37.4)
Age			
	<70	62	(36.9)
	≥70	106	(63.1)
ECOG			
	0-1	157	(93.5)
	2-3	11	(6.5)
T			
	1	3	(1.8)
	2	40	(23.4)
	3	108	(63.1)
	4	19	(11.1)
	x	1	(0.6)
N			
	0	82	(48)
	1	46	(26.9)
	2	30	(17.5)
	x	13	(7.6)
Stage			
	I	31	(18.1)
	II	51	(29.8)
	III	89	(52.1)
Site of primary tumor			
	Colon	123	(71.9)
	Rectum	43	(28.1)
Metachronous metastasis			
	No	127	(74.3)
	Yes	44	(25.7)

CRC, colorectal cancer.

Table S2. Association between miR-199b and progression (local or distant) in 171 stage I–III CRC patients.

**Stage I**	**No. Cases**	**High miR-199b (%)**		**Low miR-199b (%)**		***p***
Progression	31	27		4		0.605
No	26	23	(88.5)	3	(11.5)	
Yes	5	4	(80)	1	(20)	
**Stage II**	**No. Cases**	**High miR-199b (%)**		**Low miR-199b (%)**		***p***
Progression	51	37		14		0.663
No	42	31	(73.8)	11	(26.2)	
Yes	9	6	(66.7)	3	(33.3)	
**Stage III**	**No. Cases**	**High miR-199b (%)**		**Low miR-199b (%)**		***p***
Progression	89	70		19		0.609
No	56	45	(80.4)	11	(19.6)	
Yes	33	25	(75.8)	8	(24.2)	

Table S3. Univariate and multivariate Cox analyses in the cohort of 157 patients with early-stage CRC.

		Univariate PFS Analysis			Multivariate PFS Cox Analysis		
		**HR**	**95% CI Lower Upper**	**Significance**	**HR**	**95% CI Lower Upper**	**Significance**
Gender				0.210			-
	Male	1.000					
	Female	0.729	0.445 to 1.195		-	-	
Age				0.007			0.009
	<70	1.000			1.000		
	◎70	2.145	1.235 to 3.726		2.254	1.220 to 4.165	
Stage				0.005			0.037
	I-II	1.000			1.000		
	III	2.028	1.243 to 3.309		1.887	1.040 to 3.423	
ECOG				<0.001			<0.001
	0-1	1.000			1.000		
	2-3	2.232	1.466 to 3.181		1.976	1.352 to 2.889	
T				0.542			-
	1-2	1.000					
	>2	1.089	0.828 to 1.431		-	-	
N				0.084			-
	0-1	1.000					
	2-x	1.569	0.941 to 2.618		-	-	
SET				0.017			0.014
	Low	1.000			1.000		
	High	2.111	1.142 to 3.902		2.270	1.182 to 4.359	
MiR-199b				0.016			0.023
	Low	1.000			1.000		
	High	0.535	0.320 to 0.892		0.519	0.295 to 0.913	

PFS: progression-free survival; CI: confidence interval, HR: hazard-ratio.

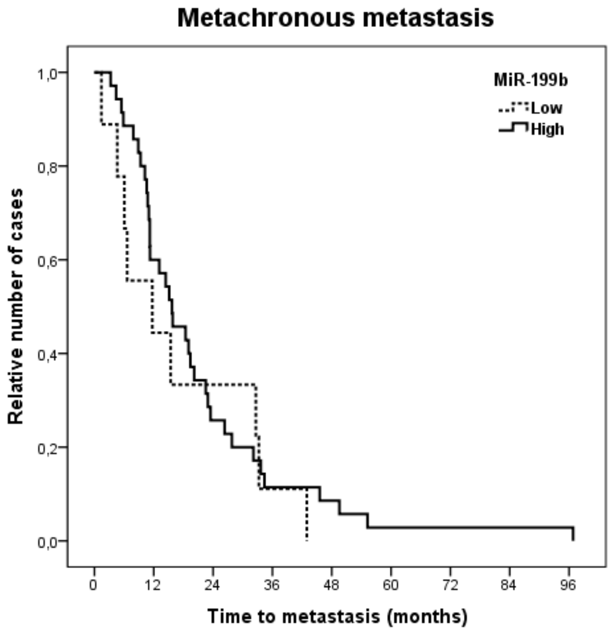

Figure S1. Clinical impact of miR-199b over time to metastasis.

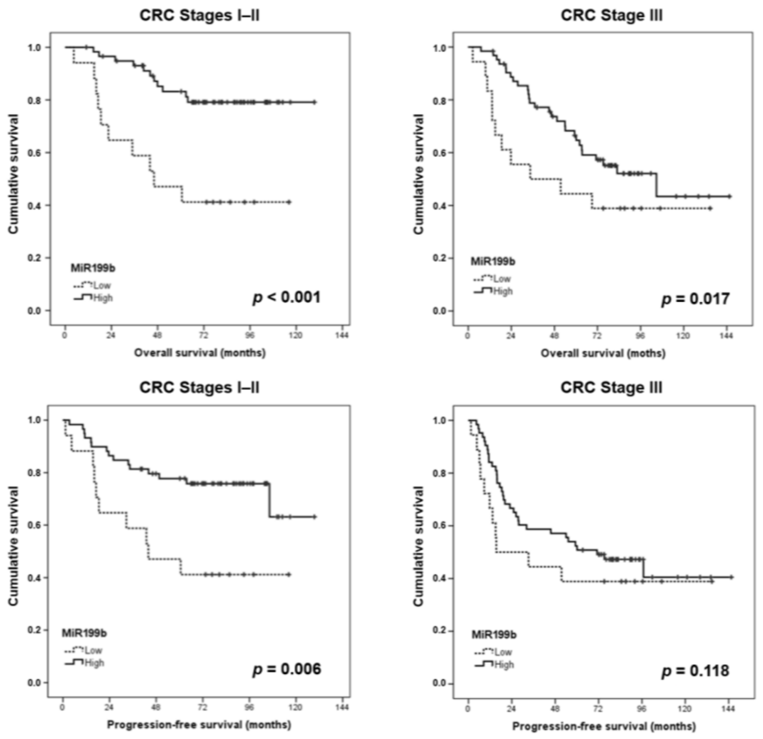

Figure S2. Kaplan–Meier analyses for miR-199b expression in the cohort of 157 CRC cases stratified by stage. CRC, colorectal cancer.

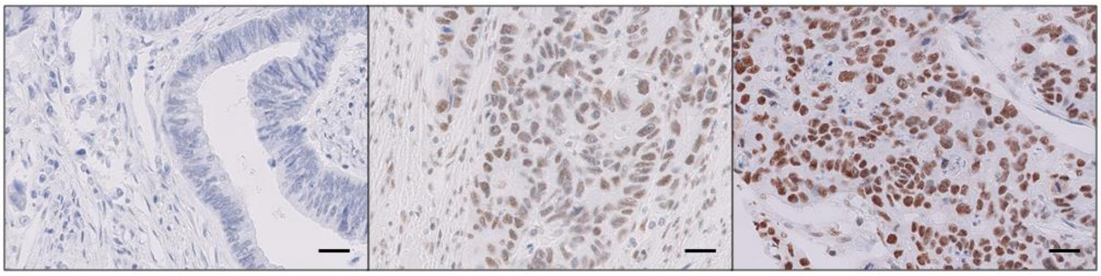

(**a**)

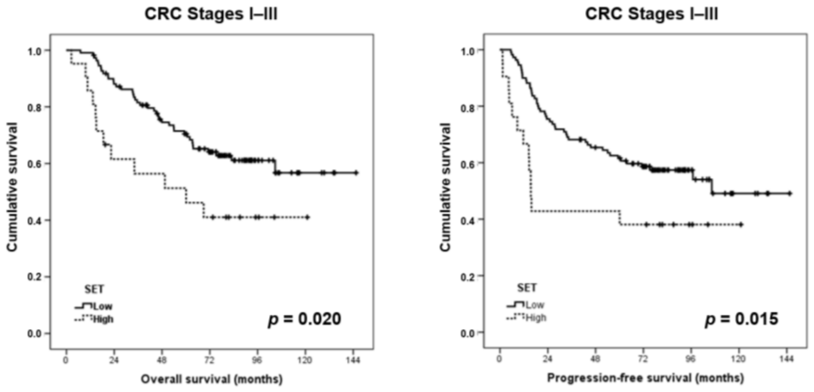

(**b**)

Figure S3. Clinical significance of SET in the cohort of 157 early-stage CRC patients. (**a**) Immunohistochemical images of SET in LARC patients representing low, medium, and high staining. The line shows 25 μm. Original magnification 400×. (**b**) Kaplan–Meier analyses for SET expression in early-stage CRC patients. SET, SET Nuclear Proto-Oncogene; LARC, locally advanced rectal cancer.
